# Identification of diagnostic markers for ASD: a restrictive interest analysis based on EEG combined with eye tracking

**DOI:** 10.3389/fnins.2023.1236637

**Published:** 2023-10-11

**Authors:** Binbin Sun, Bryan Wang, Zhen Wei, Zhe Feng, Zhi-Liu Wu, Walid Yassin, William S. Stone, Yan Lin, Xue-Jun Kong

**Affiliations:** ^1^Shenzhen Maternity and Child Healthcare Hospital, Southern Medical University, Shenzhen, China; ^2^Martinos Center, Massachusetts General Hospital, Harvard Medical School, Boston, MA, United States; ^3^Department of English and Creative Writing, Brandeis University, Waltham, MA, United States; ^4^McLean Hospital, Harvard Medical School, Belmont, MA, United States; ^5^Beth Israel Deaconess Medical Center, Harvard Medical School, Boston, MA, United States

**Keywords:** EEG, functional connectivity, eye tracking, ASD biomarker, ASD early diagnosis

## Abstract

Electroencephalography (EEG) functional connectivity (EFC) and eye tracking (ET) have been explored as objective screening methods for autism spectrum disorder (ASD), but no study has yet evaluated restricted and repetitive behavior (RRBs) simultaneously to infer early ASD diagnosis. Typically developing (TD) children (*n* = 27) and ASD (*n* = 32), age- and sex-matched, were evaluated with EFC and ET simultaneously, using the restricted interest stimulus paradigm. Network-based machine learning prediction (NBS-predict) was used to identify ASD. Correlations between EFC, ET, and Autism Diagnostic Observation Schedule-Second Edition (ADOS-2) were performed. The Area Under the Curve (AUC) of receiver-operating characteristics (ROC) was measured to evaluate the predictive performance. Under high restrictive interest stimuli (HRIS), ASD children have significantly higher α band connectivity and significantly more total fixation time (TFT)/pupil enlargement of ET relative to TD children (*p* = 0.04299). These biomarkers were not only significantly positively correlated with each other (R = 0.716, *p* = 8.26e−4), but also with ADOS total scores (R = 0.749, *p* = 34e-4) and RRBs sub-score (R = 0.770, *p* = 1.87e-4) for EFC (R = 0.641, *p* = 0.0148) for TFT. The accuracy of NBS-predict in identifying ASD was 63.4%. ROC curve demonstrated TFT with 91 and 90% sensitivity, and 78.7% and 77.4% specificity for ADOS total and RRB sub-scores, respectively. Simultaneous EFC and ET evaluation in ASD is highly correlated with RRB symptoms measured by ADOS-2. NBS-predict of EFC offered a direct prediction of ASD. The use of both EFC and ET improve early ASD diagnosis.

## Introduction

Autism spectrum disorder (ASD) is characterized by social impairment and restrictive/repetitive behaviors (RRBs). Given a rapidly rising prevalence (Maenner et al., 2021), the lack of effective treatments, and the consequent need for lifelong care for most patients (Bal et al., 2015), it is imperative to generate early predictive biomarkers (Towle and Patrick, 2016; Dow et al., 2017; Zwaigenbaum and Penner, 2018) that can identify pathways of early intervention and improved prognosis. Objective biomarkers for identifying ASD in early life have been explored in recent years (Frye et al., 2019; Clairmont et al., 2022; Prakash et al., 2023), imparting value to further endeavors to identify sensitive and specific biomarkers with high predictive value for clinical applications. RRBs are much less studied than social interaction impairments and provide a more specific hallmark feature for ASD diagnosis. Restricted interests are defined by the DSM-5 criteria as fixed interests with abnormal intensity, an advanced form of repetitive stereotyping behavior, which are essential for clinical diagnosis of ASD (Hyman et al., 2020). ASD’s another core symptom social communication deficit is probably due to its early obsessive interest in some non-social objects, which reduces its attention to social inputs and relevant information leading to social communication deficit and repetitive and stereotyped interest behavior in the later stage of ASD (Sasson and Touchstone, 2014). Studies have shown that 75% to 95% of patients with ASD have at least one restricted interest, and quite high proportion of patients have multiple restricted interests; Therefore, RRBs are good and reliable predictors of ASD outcomes and are stable by age (Troyb et al., 2016). The restricted interests are not associated with the severity of intellectual capability or other ASD co-morbid symptoms. While examining siblings with ASD, the restricted interests were found to be clustering in the family (Lam et al., 2008; Smith et al., 2009) as so called broader autism phenotype (stereotypy and apathy). These research results indicate that restricted interests as a featuring core presentation emerged from ASD trait are quite reliable early predictors of ASD. The severity of RRBs significantly impacts social functioning from early on with reward system dysfunction (Kohls et al., 2018). Like social interaction evaluation, psychometric testing for RRBs is also largely subjective and not highly reliable in the early developmental stages (Ozonoff and Griffith, 2000; South et al., 2005; Wolff et al., 2016; Hooker et al., 2019).

Functional connectivity indicates how different brain regions interact with each other, which is mainly measured by functional magnetic resonance imaging (fMRI; Emerson et al., 2017; Liu et al., 2020, 2021; Nair et al., 2021) and EEG (Bosl et al., 2018; Wilkinson et al., 2019, 2020) in ASD research. Compared with fMRI, EEG is less costly, has higher temporal resolution, and is better tolerated by young children with ASD (Bell and Cuevas, 2012; Wadhera and Mahmud, 2022). EEG functional connectivity (EFC) has been long explored as a biomarker due to its atypical findings in ASD (O'Reilly et al., 2017). Generally, there have been observed long-range functional underconnectivity (Just et al., 2004; Hughes, 2007; Wantzen et al., 2022; Geng et al., 2023), and short-ranged overconnectivity (Belmonte et al., 2004; Geng et al., 2023) or a more subtle mixture of hypo- and hyper-connectivity (Kana et al., 2014; O'Reilly et al., 2017). Dysconnectivity in ASD occurs in the posterior cingulate cortex, the precuneus, and the medial frontal gyrus (Wantzen et al., 2022), in addition to a more widespread distribution of dysconnectivity (Chen, 2022). EFC relationship with RRBs has been reported (Orekhova et al., 2014; Righi et al., 2014; Haartsen et al., 2019). Early developmental white matter tract connectivity impairment could be among the earliest markers of ASD pathology, with initial signs emerging within the first year of life (Wolff et al., 2012; Jin et al., 2015; Wolff et al., 2015). Another study showed that high-risk infants for ASD with lower frontal connectivity and higher right temporoparietal connectivity at 3 months predicted more severe ASD symptoms at 18 months (Dickinson et al., 2021). The use of EFC for diagnostic purposes has also been explored by several independent research teams (Pollonini et al., 2010; Ahmadlou et al., 2012; Duffy and Als, 2012; Khan et al., 2013; Jamal et al., 2014; Khan et al., 2015). However, a recent study reported strong EFC overlap in ASD and control subjects (Garcés et al., 2022), which warrants further investigation.

Eye tracking (ET) has been used to perform early screening of ASD (Black et al., 2017; Nyström et al., 2018; Artoni et al., 2020) and to understand how RRBs relate to attention and motivation (Sasson and Touchstone, 2014; Harrop et al., 2018; Vacas et al., 2021). Compared with TD, individuals with ASD spend more time focusing on non-social objects such as cars and computers with subtle sex differences (Harrop et al., 2017, 2018, 2020), which appears related to irregular connectivity between the limbic system and the frontal lobe (Nichols et al., 2014). These findings suggest that paying too much attention to details of non-social objects contributes to and/or reflects impaired social development.

An integrated EEG and ET approach has been explored in recent years (Billeci et al., 2017; Vettori et al., 2020; Wadhera and Kakkar, 2021; Zhang et al., 2021). However, these promising preliminary studies focused on measuring social impairment. There has been no study yet to evaluate RRBs, the largely neglected core ASD feature, with this integrated EEG and ET approach. As mentioned previously, both EFC and ET are good measures for RRBs. Using the two methods simultaneously offers an unexplored opportunity to understand their relationship further, and directly compare their use separately and in combination. Moreover, we will use a novel machine learning (ML) prediction-based extension of the preexisting network-based statistic (NBS) toolkit called NBS-Predict (Serin et al., 2021), to perform connectome based predictions using this data to help enhance the utility of these biomarkers in early ASD screening and diagnosis.

## Materials and methods

### Participants

ASD children were recruited from the Shenzhen Maternal and Child Healthcare Hospital (SMCHH), Shenzhen, China. Inclusion criteria included: (1) age 2–4 years old (this age group allows for a reliable ASD diagnosis using the gold standard, Autism Diagnostic Observation Schedule-Second Edition (ADOS-2), to compare our biomarkers to), (2) clinical diagnosis of ASD by two experienced pediatric psychiatrists according to the Diagnostic and Statistical Manual of Mental Disorders 5th Edition (DSM-5) and ADOS-2. Typically developing (TD) children were age- and sex-matched, from the same area and screened by a developmental pediatrician to exclude neuro-psychiatric disorders, including ASD or development delay. This study was approved by the Medical Ethics Association of Shenzhen Maternal and Child Health Hospital (SFYLS [2022]026), and the parents signed informed consent. De-identified data was shared with Massachusetts General Hospital (MGH) under Institutional Review Board number 2022P002152.

### Experimental equipment

The Eyelink1000plus eye tracker based on infrared tracking technology produced by SR Research and the 32-lead EGI instrument (HydroCel Geodesic Sensor Net) produced by Electrical Geodesics, Inc. (EGI) were used to collect the data synchronously. The eye tracker, EGI, and the host computer with E-prime v2.0 were set together under the same local network (Details of the hardware synchronization are available at: https://www.sr-research.com/hardware-integration).

### Experimental stimulus

**Figure 1:**
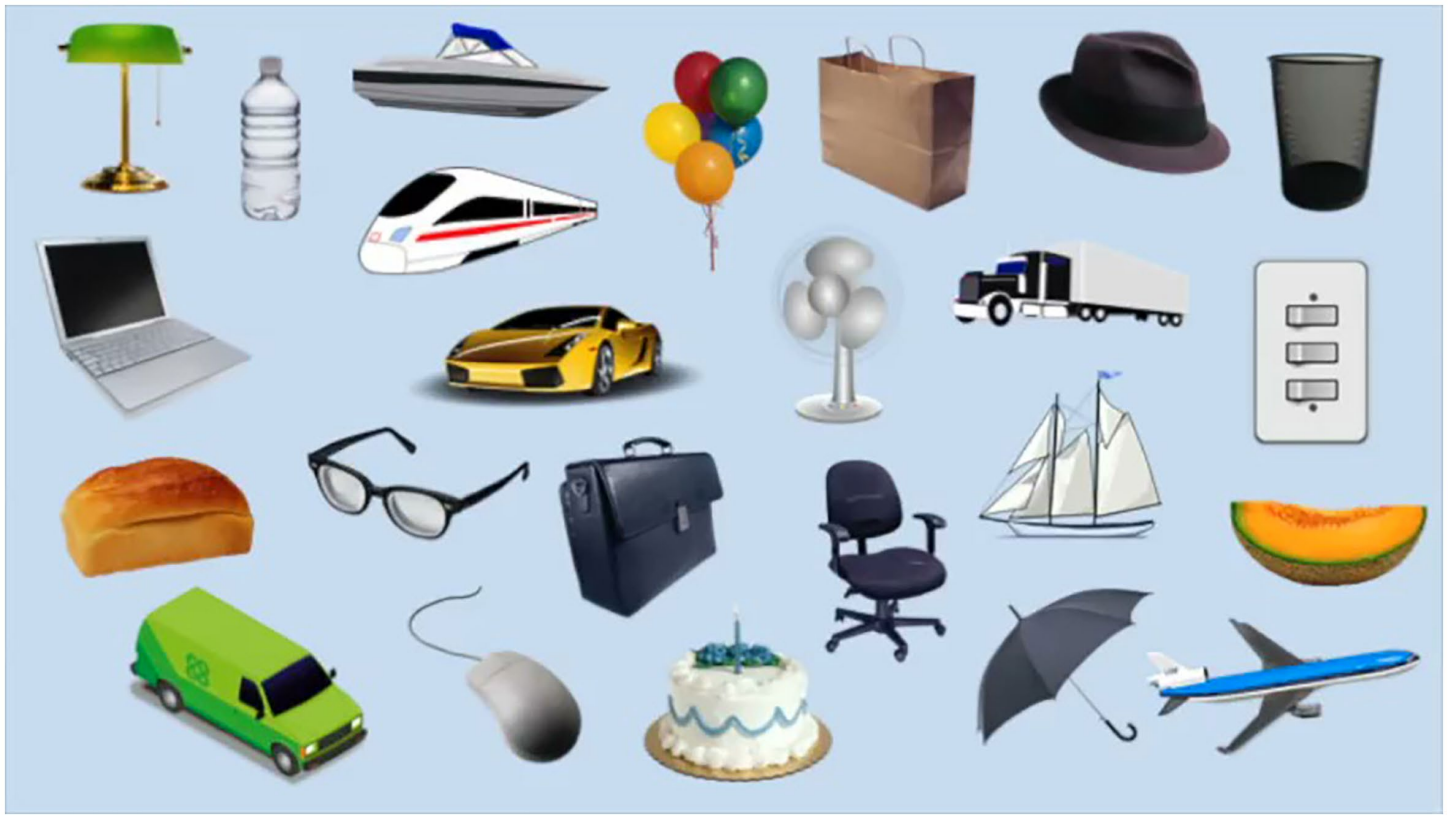
Restricted interest objects.

The restrictive interest paradigm (A figure with 24 objects randomly arranged, Figure 1) was created at MGH, based on the ASD stereotyped behavior scale and the Yale special interest interview (South et al., 2005). It includes 12 images of non-social objects such as means of transportation and electrical appliances, which are likely to appeal to individuals with “autistic” interests, identified together as “high restrictive interest stimuli (HRIS).” It also includes 12 images of neutral interest stimuli such as hats and balloons, which are defined as low restrictive interest stimuli (LRIS). Photoshop CC 2021 software^1^ was used to standardize the size (5 × 12 inch), and number of pixels (360 × 864 pixels) of all images.

### Experimental process

**Figure 2:**
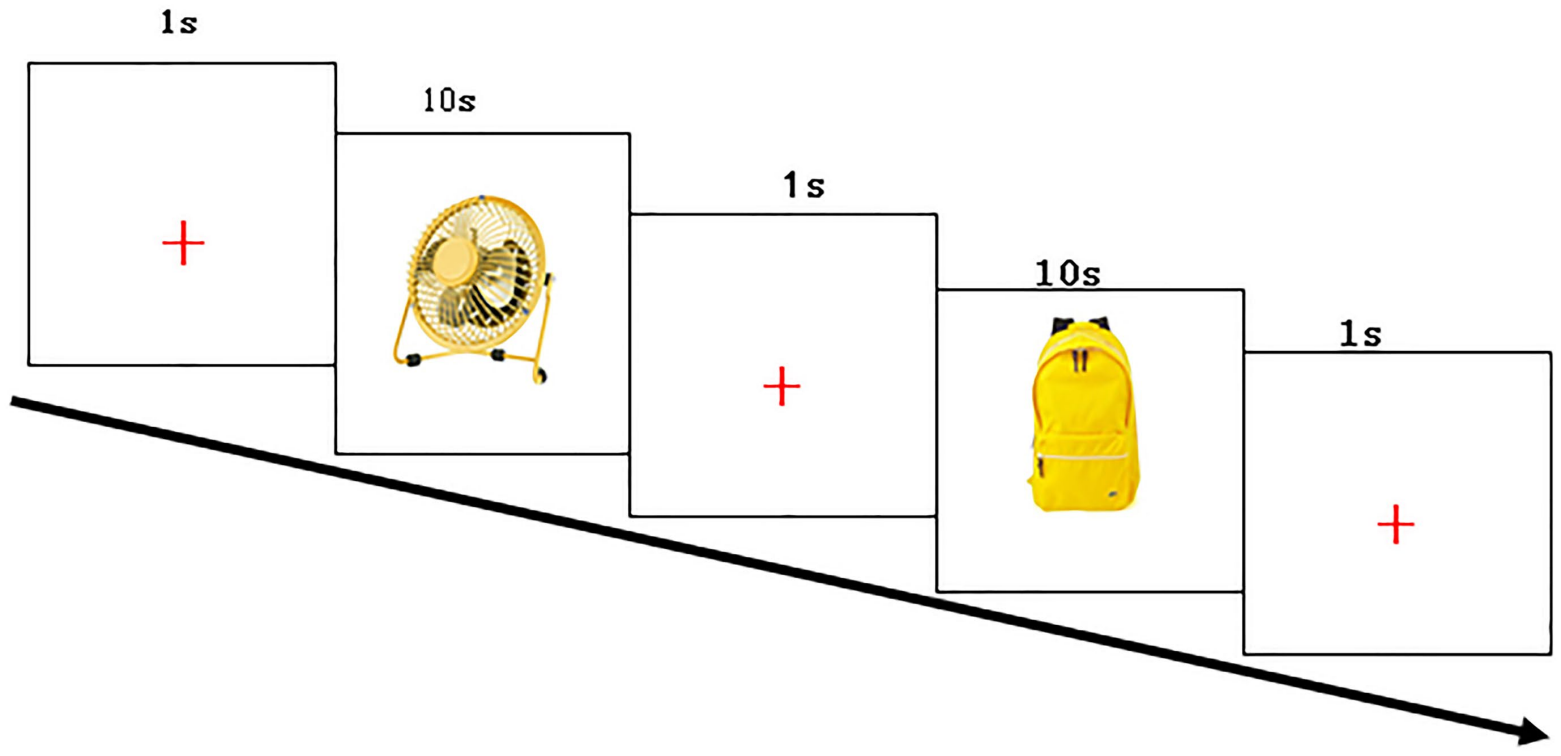
Stimulus flowchart.

The experiment was carried out in an examination room with sound insulation and constant lighting. The examiner guided the parent to hold the child during the experiments to maintain head stability. The participants were 65 cm away from the eye tracker monitor. The examiner fixed the subject’s chin on the chin rest of the eye tracker to keep the head stable, put the EGI 32 conductive electrode cap on the subject’s head, and injected saline into the electrode to render the electrode impedance less than 50 KΩ. We then adjusted the head position and collected the data. To evaluate the quality of data acquisition, we selected the sampling rate of pupil and cornea (Pupil + CR) in both eyes, so that the sampling cursor turns green at the same time, the specific parameters are as follows: Left eye: Error: < 0.5°avg., < 1°max (Poor). Right eye: Error: < 0.5°avg., < 1°max (Poor), If it is always red, it indicates that the subject’s eyes cannot focus on the “+,” and the eyes have drifted. At this time, the researchers needed to adjust the Headband of the subjects until the cursor “Pupil + CR” turns green to keep the standard state. The right eye was selected as the reference eye to start the eye tracker calibration. The examiner then guided the subjects to complete the five-point calibration of the eye tracker, after which a “+” appeared on the screen. The subjects were then asked to look at the “+” and 24 different images of objects that appeared in turn in the center of the screen. The formal experiment included 24 stimuli, in which each stimulus was displayed on the screen with yellow color and white background for 10 s, with a 1-s stimulus interval between each other (Figure 2). The total duration of the experiment was 5 min. The cursor “Pupil + CR” on the eye tracker interface remained green throughout the experiment, indicating good data acquisition quality.

### Data processing and analysis

After synchronously collecting data using EGI and ET devices, we analyzed the EEG and ET data separately and described the correlation between the two and their relationship with clinical indicators. See below for a more detailed description.

### EEG data preprocessing and analysis

**Figure 3:**
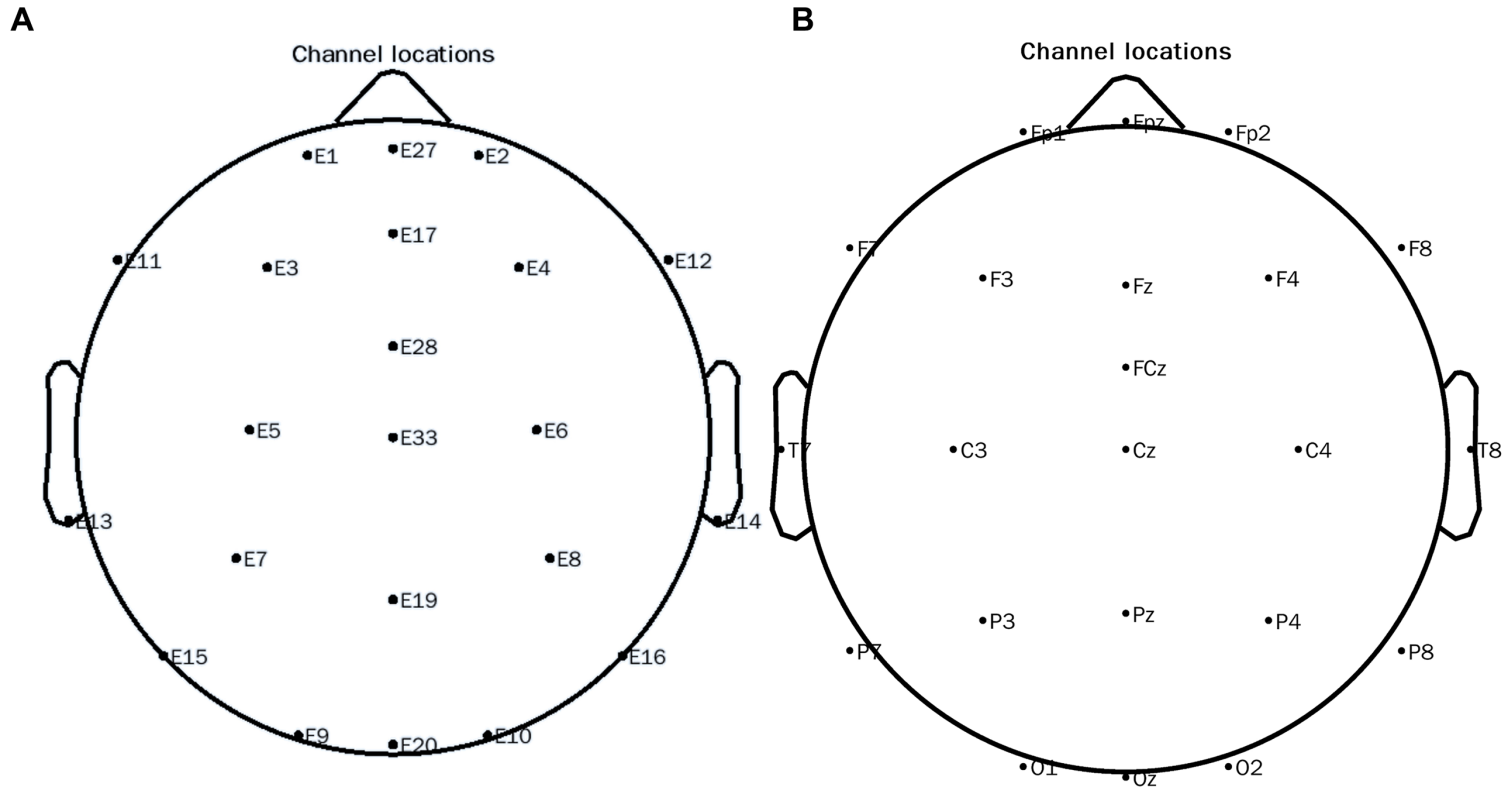
10–20 electrode distribution map of the EEG system. **(A)** Original channel locations distribution map; **(B)** Channel locations distribution map after conversion.

EEGLAB v.13.4.4b toolbox^2^ in MATLAB22b 2019 (see footnote 2) was used to process the data: The high-pass filter was set at 0.1 Hz, the cleanLineNoise function in the PREP toolkit^4^ was applied to remove 50 Hz interference and its harmonics, and the low-pass filter was set to 30 Hz. Bad channels were interpolated, followed by re-referencing to compute the average reference. We took the beginning of the stimulation as the start time, the time interval for data segmentation was −1~2 s, and baseline correction was performed followed by independent component analysis. A threshold was used to remove the segments exceeding −100~100 μV. The peripheral electrode was removed to match the 10–20 system. The distribution after pretreatment is shown in Figure 3A and the corresponding electrodes are shown in Figure 3B. After that, we used the Current Source Density (CSD) toolbox v1.1.^5^ to perform spatial Laplace transform on the signal of each electrode. Next, we used the Morlet wavelet transform to decompose the spectrum of the preprocessed data. The decomposition frequency was 4~30 Hz, and the interval of frequency points was 1 Hz. Weighted Phase-Lag Index (WPLI) was calculated by using the wavelets corresponding to the three periods of the center frequency point for the complex spectrum of all time-frequency points of each segment, the baseline was −624~−376 ms. 500~1,400 ms at alpha 8~13 Hz time and frequency ranges were chosen. When a certain cortical region is activated, the rhythmic activity of a specific frequency is manifested as a decrease in amplitude, a physiological phenomenon called event-related desynchronization “ERD.” In this experiment, the Power value in the frequency region is lower than the baseline, and it is selected as the observation variable, ERD. Two-sample t-tests and paired t-tests were used for inter-group and intra-group comparisons of the mean values of each component in the window, and NBS correction was carried out for Edge *p* = 0.005, component *p* = 0.05, and the number of permutations was 10,000 (Bockarjova et al., 2022). We also ran ML using EFC to predict ASD and TD based on the results of the WPLI index under HRIS.

We used network-based machine learning models suitable for small sample-sized data (Ren et al., 2023). The NBS-Predict toolkit (Serin et al., 2021), which combined network-based system (NBS) with ML to perform connectome-based prediction. In a cross-validation procedure, the data was split into 5 subsets, and for each iteration, one of the five subsets is selected as a test set, while the remaining subsets (4 subsets) are combined to be used as a train set, The cross-validation structure is also repeated 10 times to reduce the variation in the model performance estimation; the edges with *p*-values below a predefined value of *p* threshold (0.01) were then selected.

The indicators for evaluating model performance included AUC, Sensitivity, and specificity. A total of 5,000 permutations were generated to estimate *p*-values. A total of 3 models were constructed, Logistic Regression, Linear Support Vector Classification, Linear Discriminant Analysis, and the optimal model was then selected from the results.

### ET data preprocessing and analysis

The ET data was generated and extracted using the Eyelink1000plus’s built-in software Data View, if the missing values of fixation time (FT) were more than 30% of the total fixation time (TFT), the data were discarded. If it is less than 30%, the mean value was used in the case of symmetric data distribution (O'Brien, 2009). After preprocessing including outlier elimination and normalization, the value of FT > 100 ms was retained (Holmqvist et al., 2011), and average pupil sizes, and TFT, were used to describe the relationship with EFC during the synchronous acquisition. An independent sample t-test was performed using MATLAB22b software to compare the differences in ET between the two groups.

### Correlation analysis of EFC, ET, and ADOS-2 scores

Pearson correlations were used to evaluate the relationship between WPLI values of EFC, pupil size and TFT of ET and ADOS-2 scores, and correlation metrics values were obtained. False Discovery Rate (FDR; Benjamini and Hochberg method) was used to obtain the corrected pFDR values.

### ROC curve analysis

ROC curve analysis was performed to validate the predictive performance of ET parameters by calculating the area under the curve (AUC) and determining its optimal sensitivity and specificity compared to that of ADOS-2 total and sub-scores.

## Results

The demographic profiles of ASD and TD participants are shown in Table 1. We included a total of 32 ASD and 27 age- and sex-matched TD in this study. The maternal childbearing age and mode of delivery were also matched for both groups. ADOS-2 scores of the ASD group was 15.09 ± 3.01 indicating moderate severity.

**Table 1:** Demographic and clinical characteristics of ASD and TD.

	ASD (*n* = 32)	TD (*n* = 27)	t/χ^2^	*P*
Gender (boys/girls)	25/7	20/7	0.35	0.191
Age	3.1±0.51	2.9±0.52	0.69	0.493
Maternal childbearing age	31.40±0.61	30.01±0.20	3.12	0.893
Mode of delivery (Natural/C-section)	11/32	16/27	0.42	0.187
ADOS total score	15.09 ± 3.01	-	-	-
Social affection score	9.84 ± 3.06	-	-	-
Restricted repetitive behavior score	4.25 ± 2.16	-	-	-

ASD, Autism Spectrum Disorder; TD, Typically developing children; ADOS, Autism Diagnostic Observation Schedule, Second Edition; C, Cesarean; Values in the ASD/TD columns indicate Mean ± Standard deviation, otherwise they indicate number of subjects.

### EFC analysis results

**Figure 4:**
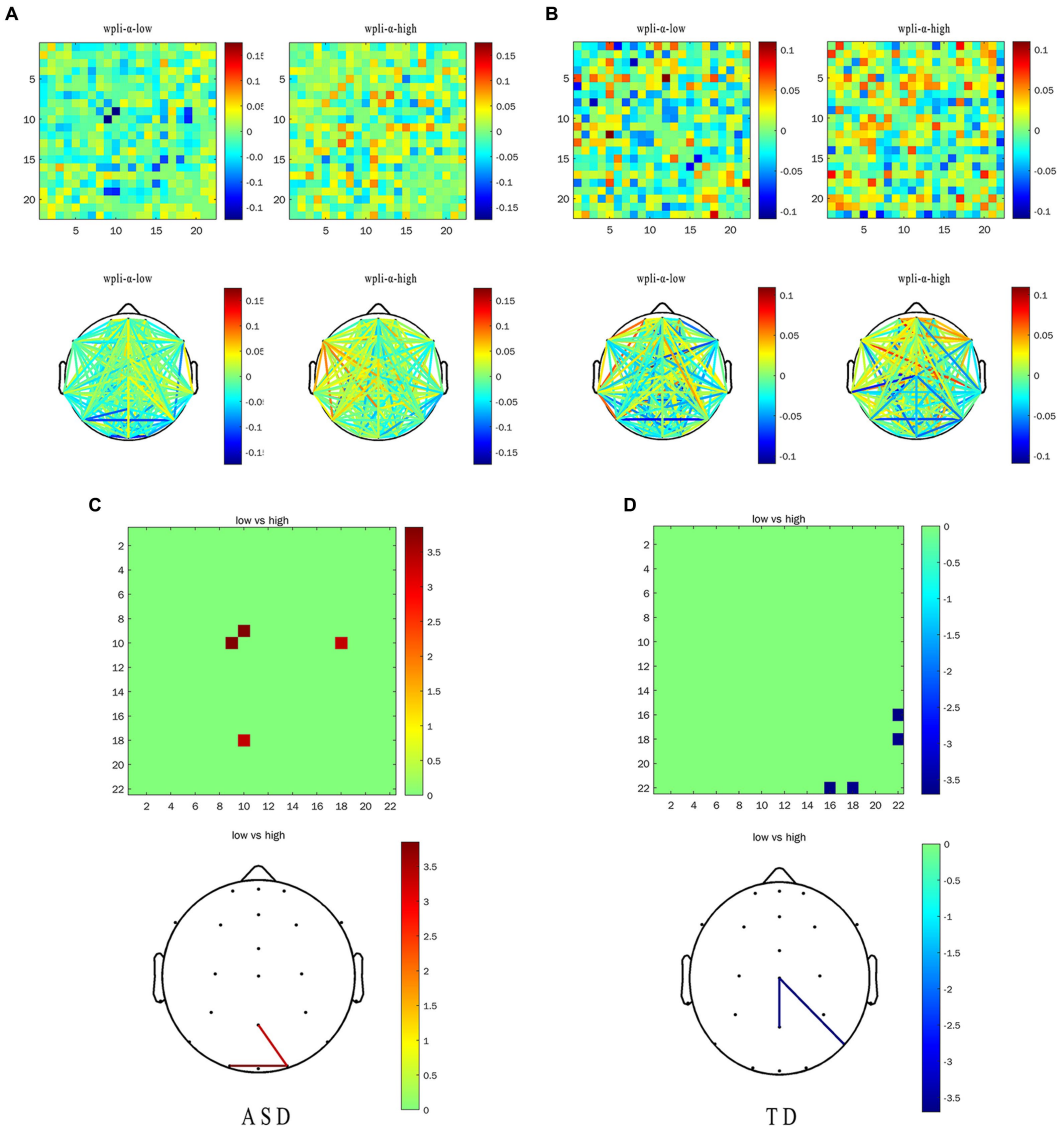
The α-band WPLI of ASD and TD under the LRIS (low) and HRIS (high). **(A)** ASD group alpha band WPLI r matrix graph and topology graph; **(B)** TD group alpha band WPLI r matrix graph and topology graph; **(C)** Brain regions with statistical difference in WPLI in ASD group; **(D)** Brain regions with statistical difference in WPLI in TD group. ASD, Autism Spectrum Disorder; TD, Typically developing children; High, high restrictive interest stimuli (HRIS); Low, low restrictive interest stimuli (LRIS). The first behavior matrix graph and the second behavior topology graph both represent the same information, and the surrounding numbers represent the energy range, which is the result graph of the functional connectivity analysis.

**Figure 5:**
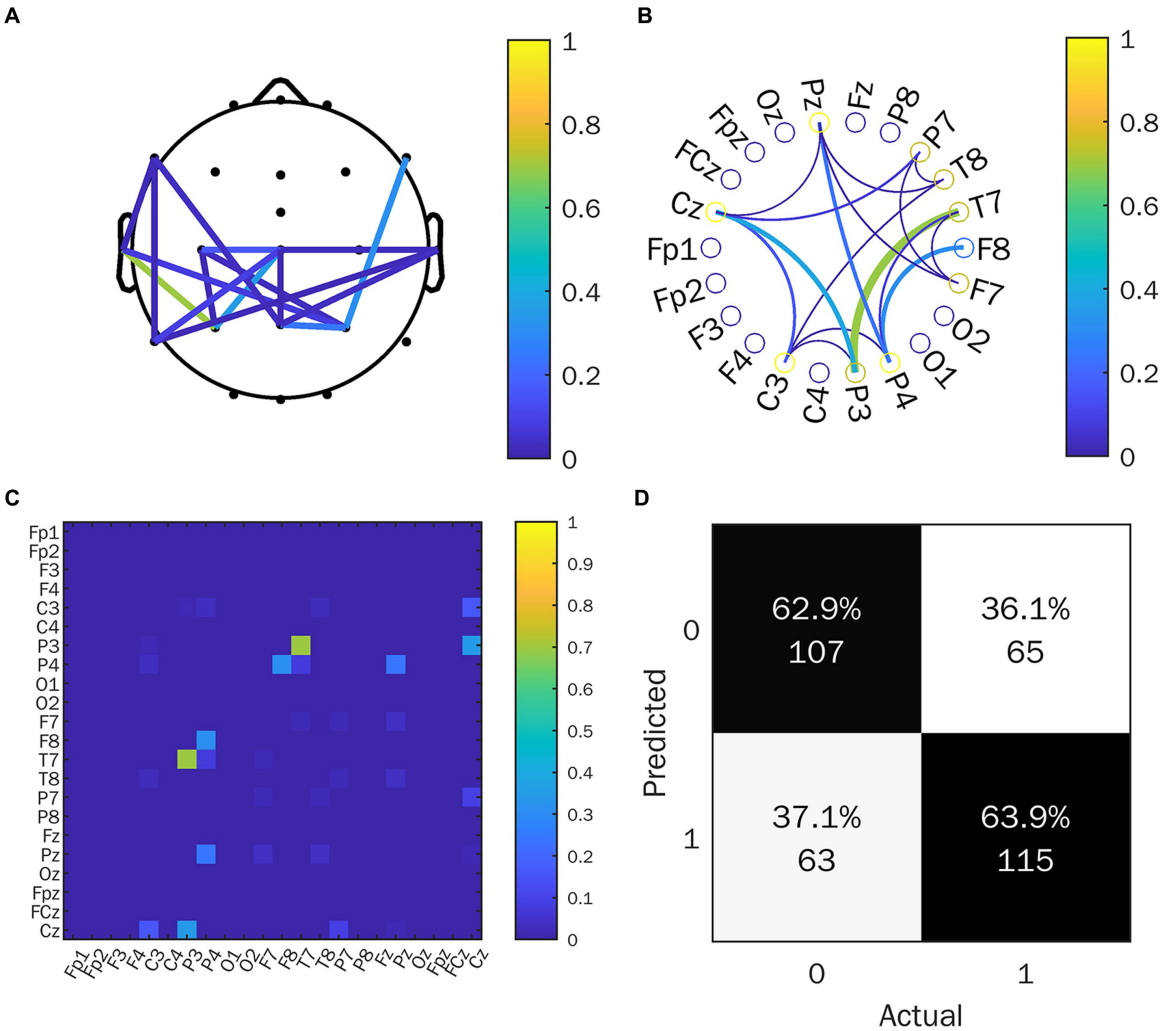
NBS-predict result. **(A)** Connected brain map; **(B)** Connected circle map; **(C)** Adjacency matrix; **(D)** Classification performance; 1 represents the ASD group, 0 represents the TD group. C, central region; P, parietal region; T, temporal region; F, frontal region; z, midline position of the brain.

We calculated the EFC index (WPLI) for all subjects, after which the data was analyzed. We mainly focused on the θ and the α frequency band average WPLI under the HRIS (high) and LRIS (low) in both ASD and TD. The significant changes were only observed in the α band. The matrix network topology diagram and NBS test results are shown in Figure 4, and the NBS-Predict results are shown in Figure 5. The ASD group had a significantly higher WPLI of α band in the parietal-occipital region under HRIS than under LRIS (t = 3.85, *p* = 0.042; Figures 4A,C). The α-band connectivity of the central parietal midline area and central posterior temporal area in TD under LRIS was significantly higher than HRIS (t = 3.70, *p* = 0.035; Figures 4B,D). The α-band WPLI index with ML (Training 80% and testing 20%; Figure 5) under HRIS showed permutation mean value was 0.600, and AUC was 0.634 (95% CI: 0.597, 0.671), sensitivity 65.1%, specificity 62.8%, for ASD from TD. The weighted network and its adjacency matrix are shown in Figures 5B,C. Under HRIS conditions, there was an imbalance in the connection between the 16 electrodes in the ASD group, the brain regions exhibiting connectivity imbalance and their corresponding degrees of electrode placement are elaborated in Table 2. Conditions with aberrant connectivity were mainly demonstrated in the central parietal area, and anterior and posterior temporal areas (Figure 5).

**Table 2:** Electrodes and their degree of connection dissonance.

Electrode	Degree
C3	4
P4	4
Pz	4
Cz	4
P3	3
F7	3
T7	3
T8	3
P7	3
F8	1

C, central region; P, parietal region; T, temporal region; F, frontal region; z, midline position of the brain.

### ET results

Under HRIS, ASD children had significantly increased TFT (*p* < 0.01) and pupil size (*p* < 0.01) relative to those in TD. In contrast, under LRIS, TD children showed significantly increased TFT (*p* < 0.01) and pupil size (*p* < 0.01) relative to those with ASD (Table 3).

**Table 3:** Comparison of ET index under two RIS in ASD vs. TD.

Interest stimulation	ET index	ASD	TD	t	*P*
High	Pupil size (μm)	4991.67 ± 155.30	2520.87 ± 350.30	4.96	*P* < 0.01
	TFT (ms)	4853.30 ± 227.43	2628.98 ± 203.61	7.28	*P* < 0.01
Low	Pupil size (μm)	2896.35 ± 57.21	4687.56 ± 208.36	−3.66	*P* < 0.01
	TFT (ms)	2205.51 ± 180.53	5914 ± 387.71	−8.83	*P* < 0.01

ET, eye tracking; RIS, restrictive interest stimuli; High, high restrictive interest stimuli (HRIS); Low, low restrictive interest stimuli (LRIS); TFT, total fixation time; ASD, Autism Spectrum Disorder; TD, Typically developing children.

### Correlation between EFC and ET index

**Figure 6:**
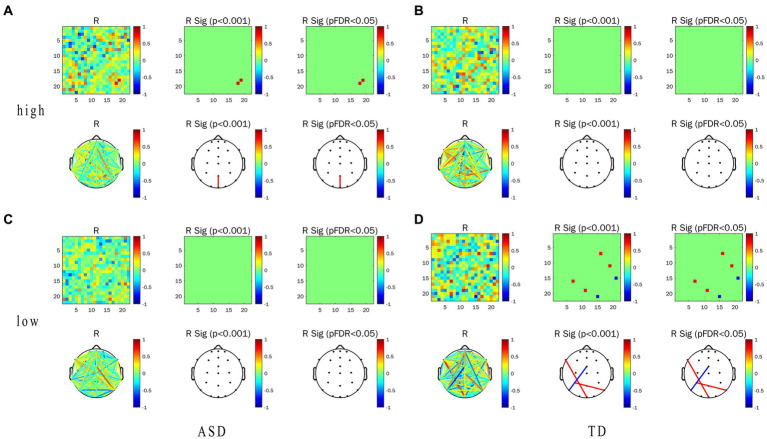
Correlation between alpha-band WPLI value and pupil size in ASD and TD under the LRIS (low) and HRIS (high). **(A)** Correlation between alpha-band WPLI value and pupil size in ASD under the HRIS; **(B)** Correlation between alpha-band WPLI value and pupil size in TD under the HRIS; **(C)** Correlation between alpha-band WPLI value and pupil size in ASD under the LRIS; **(D)** Correlation between alpha-band WPLI value and pupil size in TD under the LRIS. ASD, Autism Spectrum Disorder; TD, Typically developing children; low, low restricted interest stimuli; high, high restricted interest stimuli.

**Figure 7:**
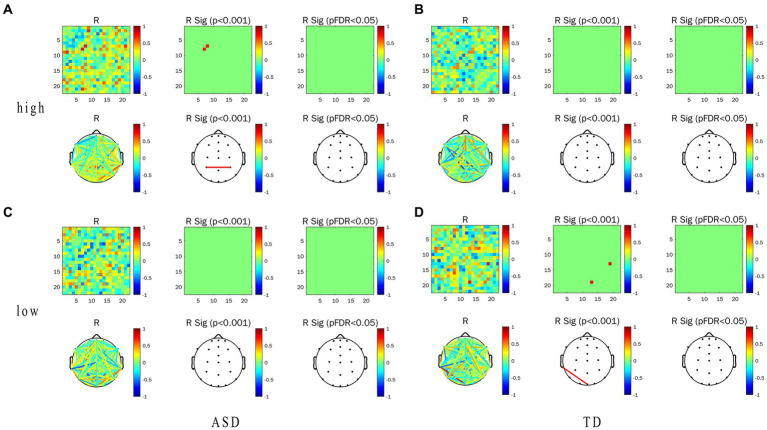
Correlation between WPLI value of α frequency band and TFT in ASD group and TD group under the conditions of LRIS (low) and HRIS (high). **(A)** Correlation between alpha-band WPLI value and TFT in ASD under the HRIS; **(B)** Correlation between alpha-band WPLI value and TFT in TD under the HRIS; **(C)** Correlation between alpha-band WPLI value and TFT in ASD under the LRIS; **(D)** Correlation between alpha-band WPLI value and TFT in TD under the LRIS. TFT, total fixation time; ASD, Autism Spectrum Disorder; TD, Typically developing children; low, low restricted interest stimuli (LRIS); high, high restricted interest stimuli (HRIS).

In this experiment, we focused on the association between the EFC and ET indicators (including pupil size and TFT) in task-state EFC in the α frequency band and their differences. Correlation heat map, network topology map, and correlation between the WPLI value and ET indicators of the two groups under the conditions of LRIS and HRIS are shown in Figure 6. for correlation with pupil size, and Figure 7 for correlation with TFT. Under HRIS the WPLI value of parietal midline -occipital midline regions in ASD was significantly and strongly positively correlated with pupil size (R = 0.806. *p* < 0.05; Figure 6A), but no significant correlations were found in TD (Figure 6B).

The WPLI value of left parietal -right parietal areas in ASD was significantly and moderately positively correlated with TFT (R = 0.716. *p* < 0.01; Figure 7A). Under LRIS, the WPLI value of the left parietal–right posterior temporal areas, left middle frontal -occipital midline areas in TD were significantly and moderately positively correlated with the pupil size (R_P3-P8_ = 0.745.R_F7-Pz_, = 0.766, *p* < 0.01). The WPLI value of the left posterior temporal-frontal central line regions in TD had a significant moderately negatively correlation with pupil size (R = −0.772, *p* < 0.01; Figure 6D). The WPLI value of left posterior temporal -occipital midline regions in TD was significantly and moderately positively correlated with TFT (R = 0.764, *p* < 0.01; Figure 7D), these correlations were not found in ASD.

### Correlation of ET and ADOS-2

**Figure 8:**
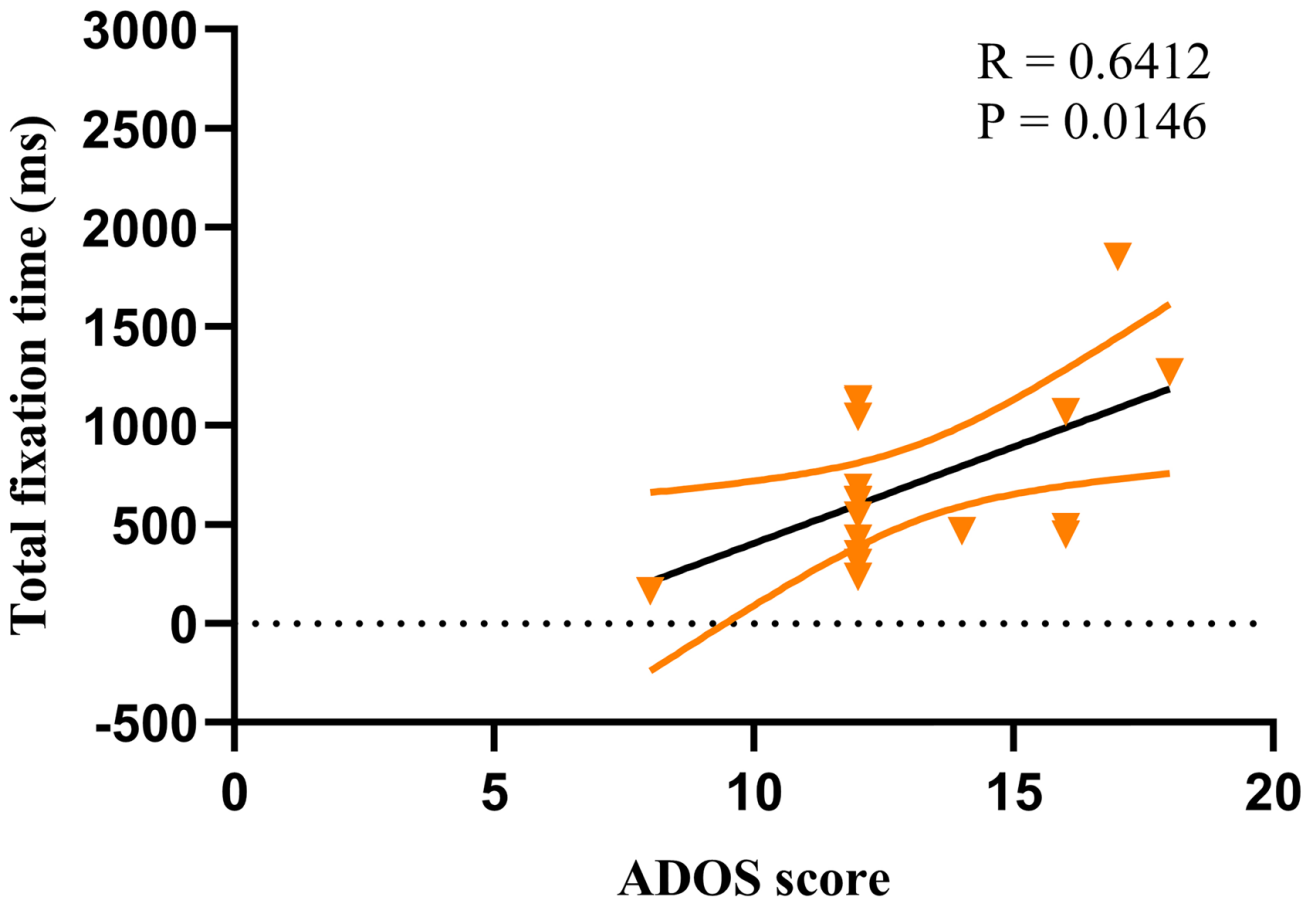
Correlation between TFT and ADOS total scores in children with ASD under HRIS (high). TFT, total fixation time; HRIS, high restricted interest stimuli; ADOS score is ADOS-2 total score.

**Figure 9:**
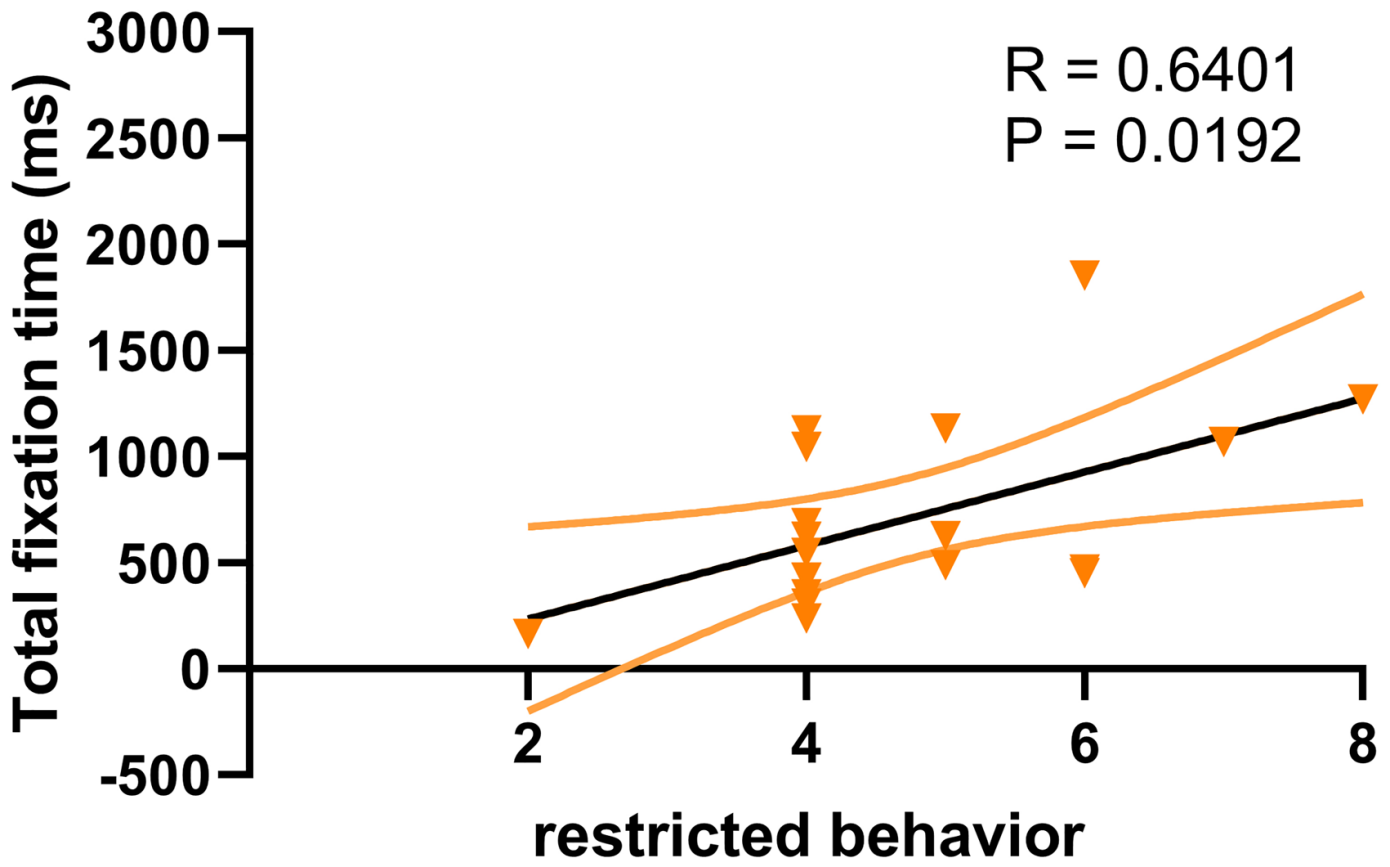
Correlation between TFT and RRB sub-scores in children with ASD under HRIS (high). TFT, total fixation time; RRB, restricted repetitive behavior; HRIS, high restricted interest stimuli; Restricted behavior is RRB sub-scores of ADOS-2.

For ASD children under HRIS, the TFT was significantly and moderately positively correlated with the ADOS total scores (R = 0.641, *p* < 0.051; Figure 8) and RRBs sub-scores (R = 0.640, *p* < 0.05; Figure 9), but not with the social affect (SA) sub-score. No such relationship was found under LRIS.

### Correlation of EFC and ADOS-2

**Figure 10:**
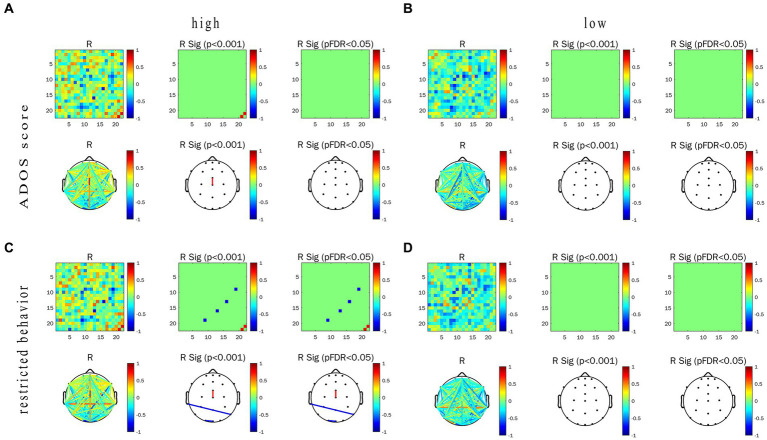
Correlations between WPLI value of α frequency band, ADOS score and restricted behavior in ASD group under the conditions of LRIS (low) and HRIS (high). **(A)** Correlations between WPLI value of α frequency band and ADOS score under the conditions of HRIS; **(B)** Correlations between WPLI value of α frequency band and ADOS score under the conditions of LRIS; **(C)** Correlations between WPLI value of α frequency band and RRBS score under the conditions of HRIS; **(D)** Correlations between WPLI value of α frequency band and RRBS score under the conditions of LRIS. Low, low restricted interest stimuli (LRIS); high, high restricted interest stimuli (HRIS). ADOS score is ADOS-2 total score, Restricted behavior is RRB sub-scores of ADOS-2.

For ASD children under HRIS, the WPLI value of α frequency band in the frontocentral area-central line area was significantly and moderately positively correlated with the ADOS-2 total score (R = 0.749, *p* < 0.01; Figure 10A) and RRB sub-scores (R = 0.770, *p* < 0.01; Figure 10C). While the α frequency band of the left middle temporal -right posterior temporal regions and left occipital-occipital midline regions was significantly and strongly or moderately negatively correlated with RRB sub-scores (R_T7–P8_ = −0.809, R_O1–Oz_ = −0.745, *p* < 0.01; Figures 10B,D). No significant association was found under LRIS.

### ROC curve

**Figure 11:**
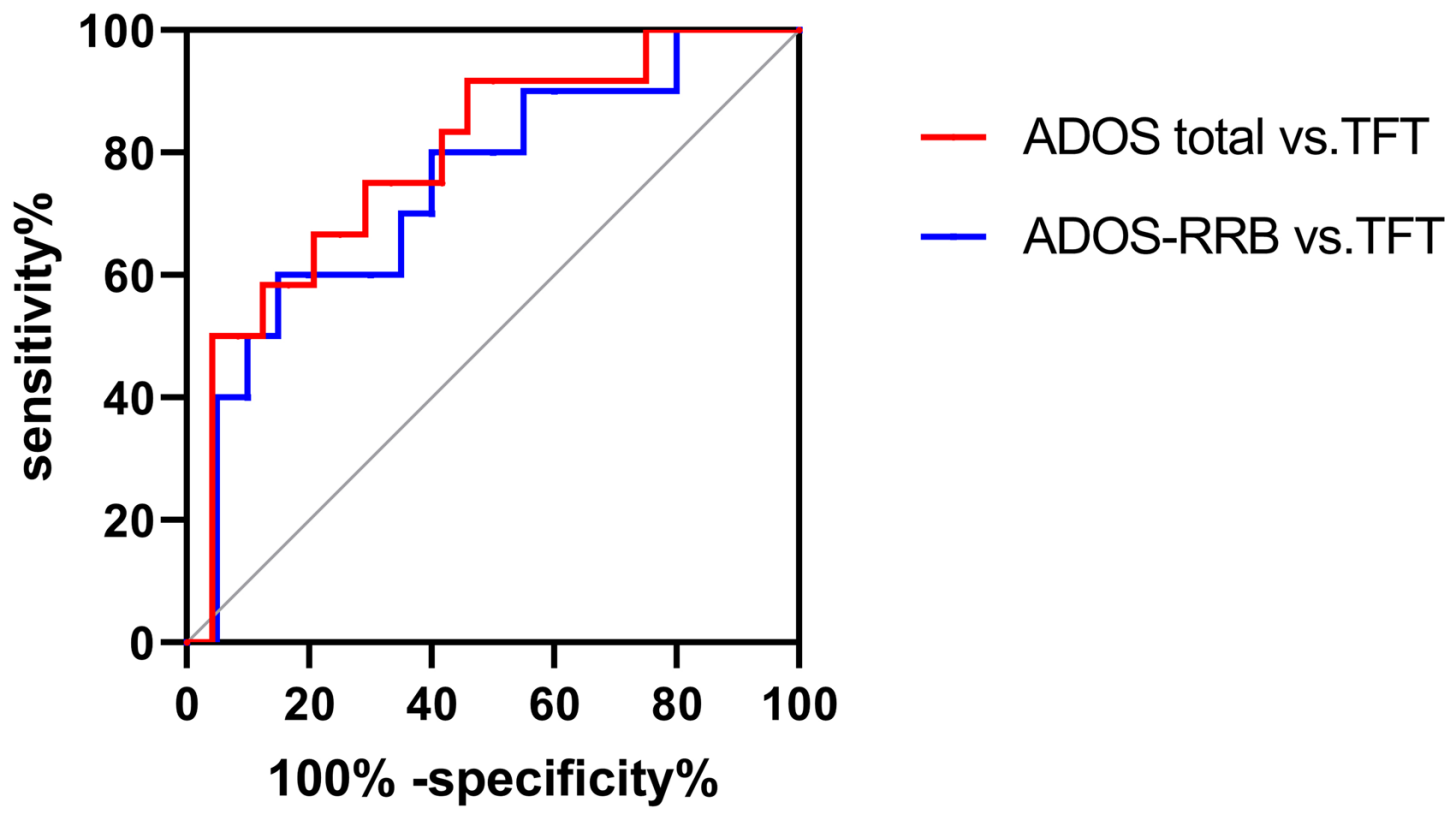
ROC curves of TFT, ADOS total and ADOS-RRB in ASD children under HRIS (high). TFT, Total fixation time; ADOS total, ADOS-2 total scores; ADOS-RRB, Restricted repetitive behavior sub-scores of ADOS-2; HRIS, high restricted interest stimuli.

The ROC curve of TFT and ADOS total, as well as RRB sub-scores under HRIS are illustrated in Figure 11. The AUC for ADOS total scores was 0.812 (95% CI: 0.608~0.975), sensitivity 91%, specificity 78.7%, with cut off 1,270 ms, for RRB sub-score it was 0.745 (95%CI: 0.524–0.966) sensitivity 90%, specificity 77.4%, with cut off 913.8 ms (Figure 11).

## Discussion

This study specifically focuses on RRBs, with a simultaneous evaluation of EFC and ET which allows a better understanding of the cognitive processing of subjects while watching restricted objects of interest. During the EFC evaluation under HRIS, ASD children showed significantly higher α frequency in the parieto-occipital region than that of LRIS (*p* < 0.05) relative to those in the TD group. Our results provide evidence in support of a strong association between EFC and RRBs and suggest its potential utilization as a biomarker to differentiate individuals with and without ASD. These results suggest further that α connectivity represents over-focused attention, and short-ranged α overconnectivity, along with long-range underconnectivity, thus supporting the Weak Central Coherence (WCC) hypothesis which accounts for the detail-focused cognitive paradigm in ASD (Happé and Frith, 2006; Serin et al., 2007). A previous longitudinal study indicated that increased α connectivity at 14 months was associated with later ASD diagnosis and dimensional variation in RRBs (Orekhova et al., 2014). The follow-up study demonstrated the association between higher α functional connectivity at 14 months and greater severity of RRBs at 36 months who met the criteria for ASD (Haartsen et al., 2019). There were differences between the two studies, however. First, they tested high-risk infants, while ours studied preschoolers, second, they used dynamic videos of spinning toys and women singing while we used sequential presentation of HRIS and LRIS images, and third, they used NBS while we used NBS-predict (Serin et al., 2021) to compute connectivity with direct prediction. Other reports in the field have been inconsistent. Alotaibi et al., for example, reported that EFC in ASD has been revealed at the theta band (Alotaibi and Maharatna, 2021), while Domínguez et al. reported increased EFC in toddlers with ASD across alpha, theta, and delta (García Domínguez et al., 2013). Boersma et al. found no differences between 2 to 5-year-old toddlers with ASD and TD in EFC over broadband (1–30 Hz) or theta-alpha band (Boersma et al., 2013). This was similar to the findings of Buckley et al. (2015).

Our study demonstrated that, under the HRIS, ASD children show significantly increased pupil size and TFT compared to TD children. While, under the LRIS, TD children show significantly increased pupil size and TFT compared to those in ASD. These results are consistent with previous research for TFT (Helminen et al., 2017; Wan et al., 2019; Kong et al., 2022) and pupil size (Lynch et al., 2018; Nyström et al., 2018; Artoni et al., 2020) explored as objective biomarkers. Individuals with ASD exhibit prolonged first fixation time on non-social HRIS, such as transportation, and demonstrate heightened attention to detail in computer and car games (Nichols et al., 2014; Harrop et al., 2018). The dilated pupil corresponds to emotional arousal (Ming et al., 2005) and ASD individuals have significantly smaller baseline pupil size (Martineau et al., 2011). Pupil dilation metrics correlate with individual differences measured by the Social Responsiveness Scale (SRS), a quantitative measure of autism traits (DiCriscio and Troiani, 2017).

EEG functional connectivity as a non-invasive method measures the electrical activity among different regions of the brain in children with ASD to understand the information exchange and mutual influence (Hull et al., 2017). By analyzing EEG signals, specific features related to ASD can be identified, which provides useful information for revealing the neural mechanisms of ASD. The lack of consistency across findings of EFC may depend on age, task, and length of the EEG recordings, the frequency band of interest, the selected index of EFC, and sample sizes, among others (O'Reilly et al., 2017; Schwartz et al., 2017). More importantly, ASD is a highly heterogeneous disorder with diverse etiology, phenotype, and functional quantity. Therefore, the results of EEG functional connectivity studies in individuals with ASD may be influenced by various factors such as age, gender, intelligence quotient, clinical symptoms, and genetic background. The functional connectivity results in children with autism may also change with different stimuli and environments. Additionally, it is important to explore the neurocognitive functional development of children with ASD through studying their EEG developmental trajectory. Our future research will continue to explore different analysis methods and indicators. For example, advanced signal processing techniques can be used to reduce the influence of noise, and more comprehensive brain activity information can be captured through multi-channel EEG recordings. Combined with functional magnetic resonance imaging (fMRI), we will further validate the reliability and effectiveness of functional brain connectivity in children with ASD (Sato and Uono, 2019), and explore its potential clinical applications.

For the correlation between EFC and ET parameters, we observed a significant and positive correlation in ASD between α band connectivity under HRIS and increased pupil size and TFT. However, no such significant correlation was found in TD. These findings demonstrate a robust association between EFC and ET parameters when measuring RRB. The combination of using EEG or ET has many advantages compared with using them alone: First, eye blinks and eye movements have significant influence on EEG signals and their subsequent analysis, simultaneous EEG and ET allow researchers easily identify, manage and suppress these artifacts to assure the quality of test reports and their interpretations. Secondly, EEG and ET simultaneous use with the same subjects and paradigms in the experiment can effectively reduce the variations of individuals and paradigm with separate recordings. Third, real-time gaze-related tasks can be performed with the combination methods, this case, the stimuli are presented only when the participant is fixating on a central object. Lastly, both ET and EEG technology have a high temporal resolution (millisecond level), and ET technology also has a high spatial resolution (<1° viewing angle), so the combination of the two can accurately reflecting the time course of cognitive processing and its neural mechanisms. ET deduces the relevant cognitive processing basis by recording the two basic ET phenomena of saccade and fixation (Rayner, 1998), while EEG explores the time process of each cognitive processing stage by recording the EEG signals associated with stimulus events and locked in time with stimulation (Luck, 2014), which can reveal the neural mechanism of each cognitive processing stage (Baccino and Manunta, 2006). The combination of the two can better understand the cognitive processing patterns and mechanisms of participants when viewing restricted objects of interest. As mentioned earlier, there have been a few studies reported using combined EEG and ET (Billeci et al., 2017; Vettori et al., 2020; Zhang et al., 2021). Their focus was on social impairment measurements, none of those studies tested RRB as we demonstrate in this work. Zhang et al. (2021) did not find an association between the original biosignals of both modalities and did not demonstrate the direct comparison as we illustrate here. Instead, they used a big data-driven deep learning Graph Convolutional Networks model, which requires hardware accelerators and takes a long time to complete. This is not practical in a typical clinical setting. The NBS-predict program we chose is a fast and convenient tool, and is suitable for using modest-sized data sets, independent of any specific hardware, by combining ML model with connected components in a cross-validation structure, which is important to avoid overfitting. Additionally, they did not evaluate the clinical parameters, which discounts the value of a clinically relevant translational research component. We propose that a combination of both evaluation measures to study RRB features will increase sensitivity and specificity in early ASD screening, which furthers our understanding of the underlying mechanism and neural pathways in the autistic brain.

Lastly, the associations identified through the use of ADOS-2 provided more insight into our evaluation. The ROC curve of TFT demonstrated good sensitivity and specificity. For the EFC, we found that under HRIS, α frequency band of the frontal central region in ASD was significantly and positively correlated with ADOS total score and RRB sub-scores. α frequency band of the parietal–temporal-occipital (PTO) region is significantly and negatively correlated with RRB sub-scores. No significant association was found under LRIS. ADOS-2 is well recognized as a gold standard diagnostic tool for ASD. The strong associations with ADOS-2 total score and RRB sub-scores demonstrate that both TFT of ET and α band EFC around the frontal central region are reliable biomarkers and correlated with ASD severity, particularly RRB features, although the EFC showed higher association with ADOS than TFT. The previous work of Haartsen et al. (2019) demonstrated that higher global connectivity correlates with RRBs at 14 months and more severe social and communication symptoms measured by the Autism Diagnostic Interview–Revised (ADI-R) at age 3 years. No such associations with symptoms were measured on the ADOS–Generic (ADOS-G). Their replication study (Haartsen et al., 2019) found a significant correlation between higher EFC over fronto-central regions instead of global connectivity at 14 months, and more severity of RRBs measured by ADI-R at age 3 years. No such correlation was observed in ADOS-2. ET was not performed in any of those studies.

Previous reports demonstrated inconsistent EFC findings and related factors as mentioned above. We believe that analyzing the global brain connections only may not be enough to reflect the complexity of brain activity, since brain connections in ASD may differ from one participant to another. The concept of large-scale brain networks indicates that these connections are related to the interaction between different brain regions (Shafritz et al., 2008; Bressler and Menon, 2010; Di Martino et al., 2014; Nomi and Uddin, 2015; Ypma et al., 2016; Duan et al., 2017; Demetriou et al., 2018). Padmanabhan et al. believe that the aberrant key brain regions of the default mode network may underlie the neural basis of rigid thinking and poor theory of mind, which leads to atypical social interactions, and proposes that the brain areas related to the theory of mind play an important role in social and speech perception (Padmanabhan et al., 2017).

The α frequency band in the frontocentral region exhibited a significant and positive correlation with both ADOS total score and RRB sub-scores, which may reflect the heightened connectivity of the frontocentral area in ASD resulting from HRIS engagement. This finding could be linked to intentional causation as reported by Redcay (2008). This study demonstrates that EFC from the left middle-temporal area to the right posterior temporal area in ASD was negatively correlated with RRB sub-scores, which indicated a reduced development of social function when individuals with ASD paid too much attention to HRIS. This may indicate that the long-distance connectivity in the temporal region of ASD children is weakened, when excessively fixated on HRIS, thereby affecting the development of their social communication skills. This is consistent with the study of Pantelis et al. (2015), which showed reduced activity of the superior temporal gyrus associated with poor response to social situations in ASD. We also found that RRB has a negative correlation with the left occipital area, which may indicate that RRB affects emotional and social-cognitive development. Our results demonstrate that local brain activations in ASD are functionally and structurally specific, and interconnected, which may provide a neural basis for ASD deficits in theory of mind. Compared with EFC, ET is a more easily implemented and interpreted measure especially more adoptive for the subjects. In the process of ET operation, the subjects can speak if they keep their eyes on objects of interests; It also provides a clear cut-off score, which could be better for screening purposes. However, during the EEG monitoring, the subjects are not allowed to speak or move their heads because speaking or moving head will affect the quality of EEG values, which are difficult for a 2-year-old child.

This study has several limitations. First, our modest sample size limits our ability to evaluate subgroup differences such as sex, the relationship between restricted interests and gender is not clear, and the differences in restricted interests among individuals with ASD are also a potential research area between males and females. Research has shown that the restricted interests in female patients with ASD are less frequent and more difficult to observe compared with male subjects. The low prevalence could be partially related to limitation of current measurement tools (Frazier et al., 2014). In this study, the restricted interests of children with ASD follow traditional gender boundaries, and the restricted interest stimuli used are more suitable for males (such as airplanes and trains), similar to the stimuli used in other studies (Anthony et al., 2013; Cho et al., 2017; Sutherland et al., 2017), which cannot truly reveal the differences between male and female autistic patients. This also limited us from implementing an integrated model approach, such as is possible with larger datasets. In addition, this has restricted our ability to set aside a sample for external validation of our ML classification model. Future studies with larger sample sizes are needed to further investigate these questions. Second, the subjects are homogeneously Chinese. More diverse studies are needed across different ethnic backgrounds to have a wider extrapolation of our findings. Third, the subjects are 2–4 years old with limited attention spans which might impact our results, especially the EFC. Lastly, this study specifically evaluates the RRB features of ASD. In the future, we will consider evaluating social function with this combined EEG and ET approach, using a more dynamic paradigm, or conducting a longitudinal study, which would have an additional impact on the overall ASD early diagnosis.

## Conclusion

Despite these limitations, this study provides novel and promising evidence of the simultaneous use of EFC and ET in response to HRIS. These two measures were found to be complementary biomarkers for ASD early diagnosis in measuring RRBs features which have been less studied than social impairment features. The NBS-predict for EFC offered a direct prediction of ASD while ET demonstrated better prediction of ASD with higher sensitivity and specificity.

## Data availability statement

The original contributions presented in the study are included in the article/supplementary material, further inquiries can be directed to the corresponding authors.

## Ethics statement

This study was approved by Medical Ethics Association of Shenzhen Maternal and Child Health Hospital (SFYLS [2022]026) and the parents signed informed consent. De-identified data was shared with Massachusetts General Hospital (MGH) under Institutional Review Board number 2022P002152. The studies were conducted in accordance with the local legislation and institutional requirements. Written informed consent for participation in this study was provided by the participants’ legal guardians/next of kin. Written informed consent was obtained from the individual(s), and minor(s)’ legal guardian/next of kin, for the publication of any potentially identifiable images or data included in this article.

## Author contributions

BS and X-JK: conceptualization, resources, and project administration. BS: methodology, software, validation, formal analysis, and data curation. BS, ZW, ZF, and Z-LW: investigation. BS, BW, and X-JK: writing: original draft preparation. BS, X-JK, BW, WY, WS, and YL: writing: review and editing. BS and BW: visualization. X-JK: supervision. BS, YL, and X-JK: funding acquisition. All authors contributed to the article and approved the submitted version.

## Figures and Tables

**Table:** 

EEG	Electroencephalography
EFC	Electroencephalography functional connectivity
ET	Eye tracking
ASD	Autism spectrum disorder
RRBs	Restricted and repetitive behavior
TD	Typically developing children
NBS	Network-based statistic
ADOS-2	Autism Diagnostic Observation Schedule-Second Edition
AUC	The area under the curve
HRIS	High restrictive interest stimuli
LRIS	Low restrictive interest stimuli
TFT	Total fixation time
FT	Fixation time
fMRI	functional magnetic resonance imaging
DSM-5	Diagnostic and Statistical Manual of Mental Disorders 5th Edition
MGH	Massachusetts General Hospital
ICA	Independent component analysis
CSD	Current source density
WPLI	Weighted phase-lag index
FDR	False discovery rate
ROC	Receiver operating characteristic curve
WCC	Weak central coherence
DMN	Default mode network
SN	Salience network
ECN	Executive control network
ML	Machine learning

## Footnotes

^1^https://www.adobe.com/cn/products/photoshop.html

^2^https://sccn.ucsd.edu/eeglab/index.php

^4^https://vislab.github.io/EEG-Clean-Tools/

^5^http://psychophysiology.cpmc.columbia.edu/Software/CSDtoolbox
